# At the end of a two-year follow-up elevated TSH levels normalize or remain unchanged in most the children with subclinical hypothyroidism

**DOI:** 10.1186/1824-7288-36-11

**Published:** 2010-01-30

**Authors:** Filippo De Luca, Malgorzata Wasniewska, Giuseppina Zirilli, Tommaso Aversa, Teresa Arrigo

**Affiliations:** 1 Department of Pediatrics, University of Messina, Italy; 2 Dipartimento di Scienze Pediatriche Mediche e Chirurgiche, Policlinico Universitario, Via Consolare Valeria, 98123 Messina, Italy

## Abstract

Data about the natural evolution of subclinical hypothyroidism (SH) in pediatric age are very scanty. Moreover all the available reports in both aged and young patients were based on unselected study populations including also patients with either thyroid disorders or other pathological causes that are well known to be able to affect SH development and evolution. Aim of the study by Wasniewska et al was to prospectively evaluate for the first time the natural course of SH in children and adolescents with no underlying diseases and no risk factors that might interfere with the progression of SH. On the basis of the 2-year follow-up results, the Authors concluded that: a) the natural course of TSH values in a pediatric population with idiopathic SH is characterized by a progressive decrease over time; b) the majority of patients (88%) normalized or maintained unchanged their TSH; and c) TSH changes were not associated with changes of either FT4 values or clinical status or auxological parameters.

Study design of this study is very accurate and the results are robust, thus supporting the Authors' conclusions.

## Introduction

Aim of the present Commentary article is to discuss a paper concerning the natural course of idiopathic subclinical hypothyroidism (SH) in childhood and adolescence, that has been very recently published [1] and was included in the 2009 Pediatric Endocrinology Year Book [2]. Soon after its publication that paper was analyzed and commented by another Author on another journal [3].

SH is a condition of moderate thyroid failure characterized by normal circulating levels of thyroid hormones with mildly elevated TSH serum concentrations. SH is a common clinical problem in adulthood and elderly, with an average worldwide prevalence that has been reported to be in the range of 4-10% in large general population screening surveys [4] and 7-26% in studies of elderly [5]. In pediatric age SH prevalence seems to be distinctly lower than in old people, although there are only few epidemiological studies concerning childhood and adolescence [6-8]. According to one of them, SH frequency in adolescents is slightly lower than 2% [8-15].

Data from the literature regarding the natural course of SH are very controversial, probably due to the fact that most the available longterm studies are retrospective. Moreover all the available reports on the spontaneous evolution of SH in both aged and young patients have been based, to now, on unselected study populations including also patients with either thyroid disorders or other pathological causes that are well known to be able to affect SH development and evolution. The risk of progression to overt hypothyroidism, in fact, is known to be greater in those patients with underlying thyroid diseases [9] and the unfavourable prognostic value of goiter and thyroid autoantibodies was confirmed even in children and adolescents [10]. However, according to one of the few available follow-up studies on juvenile SH, this may be a benign and remitting process with a very low risk of evolution towards frank hypothyroidism [11].

In their multicenter study Wasniewska et al [1] have prospectively evaluated for the first time the natural course of SH in children and adolescents with no underlying diseases and no risk factors that might interfere with the progression of SH.

## Discussion

Clinical status, thyroid function, and autoimmunity were prospectively evaluated at entry and after 6, 12, and 24 months in 92 young patients (mean age 8.1 ± 3.0 years) with idiopathic SH.

Diagnosis of SH was based on the finding of at least two consecutive measurements of TSH serum levels between 5 and 10 μIU/ml, in the presence of normal FT4 concentrations. In the entire study population all the etiological causes of SH had been preliminarily excluded at the time of admission.

During the study mean TSH levels showed a trend towards a progressive decrease while FT4 levels remained unchanged. Overall, 38 patients normalized their TSH (group A): 16 patients between 6 and 12 months, and 22 patients between 12 and 24 months. Among the remaining 54 patients, the majority maintained TSH within the baseline values (group B), whereas 11 exhibited a further increase in TSH above 10 μIU/ml (group C). Baseline TSH and FT4 levels were similar in the patients who normalized TSH, compared with those with persistent hyperthyrotropinemia. Even in the patients of group C, both TSH and FT4 at entry were not different with respect to those of group A and B. No patients showed any symptoms of hypothyroidism during follow-up and no changes in both height and body mass index were observed throughout the observation period.

The Authors concluded that: a) the natural course of TSH values in a pediatric population with idiopathic SH is characterized by a progressive decrease over time; b) the majority of patients (88%) normalized or maintained unchanged their TSH; c) TSH changes were not associated with changes of either FT4 values or clinical status or auxological parameters; and d) TSH determination has no reason to be part of the routine check up in children, apart from specific protocols.

## Conclusions

SH is a very topical problem, which has been frequently discussed in the last few years by Consensus expert panels in order to define well-established guidelines for medical practice and treatment [12-14]. Moreover, guidelines for SH therapy were frequently discussed in the last years in a high number of editorials, commentaries, controversies or letters to the editor. With regard to SH treatment, the strategy that is shared by the majority of experts is to treat individuals with TSH which is repeatedly higher than 10 μIU/ml. When TSH is lower than 10 μIU/ml and repeatedly between 4.5 (in adults) or 5.5 μIU/ml (in prepubertal children), L-T4 therapy is to be considered only in subjects with positive anti-thyroid antibodies and/or hypothyroid symptoms or signs, whereas the subjects with idiopathic and asymptomatic SH should only be checked and periodically retested [15] a strategy may be considered as appropriate even at the light of the results of the study by Wasniewska et al, who have clearly demonstrated, through a 2-year follow-up, that in the majority of children with idiopathic SH elevated TSH levels spontaneously normalize or remain unchanged over time [1]. Due to its prospective design and the prolonged follow-up, that study was unique in the context of the studies on juvenile SH, as also underlined by Mazzaferri in his commentary (3). The patients were carefully screened for other causes of SH, including false negative congenital hypothyroidism. None of the patients had any clinical signs or symptoms during the entire follow-up. Moreover, none of the patients showed any symptoms of hypothyroidism during follow-up, and there were no significant changes in both height and body mass index throughout the observation period. The Authors of that study concluded that the natural course of TSH elevations in children and adolescents with idiopathic SH is characterized by a progressive decrease over time and that the majority of patients have a normalization of serum TSH within a 2-year follow-up.

To sum up, we agree with Mazzaferri (3) on the fact that the data in the study by Wasniewska et al (1) are robust, thus supporting the Authors' conclusion that TSH determination has no reason to be part of the routine check-up in children and adolescents, except for specific protocols.

## List of abbreviations

SH: subclinical hypothyroidism.

## Competing interests

The authors declare that they have no competing interests.
